# Supplementary material for: 5-Azacytidine-Mediated Modulation of the Immune Microenvironment in Murine Acute Myeloid Leukemia
Source: Cancers (Basel). 2022 Dec 25;15(1):118. doi: 10.3390/cancers15010118 (PMC9817798; doi:10.3390/cancers15010118)
Supplement: Supplementary file 1 [file cancers-15-00118-s001.zip › cancers-2078516-supplementary.pdf]

cancers

MDPI

Supplementary Materials

5-Azacytidine-Mediated Modulation of the Immune Microenvironment in Murine Acute Myeloid Leukemia

Nancy D. Ebelt and Edwin R. Manuel

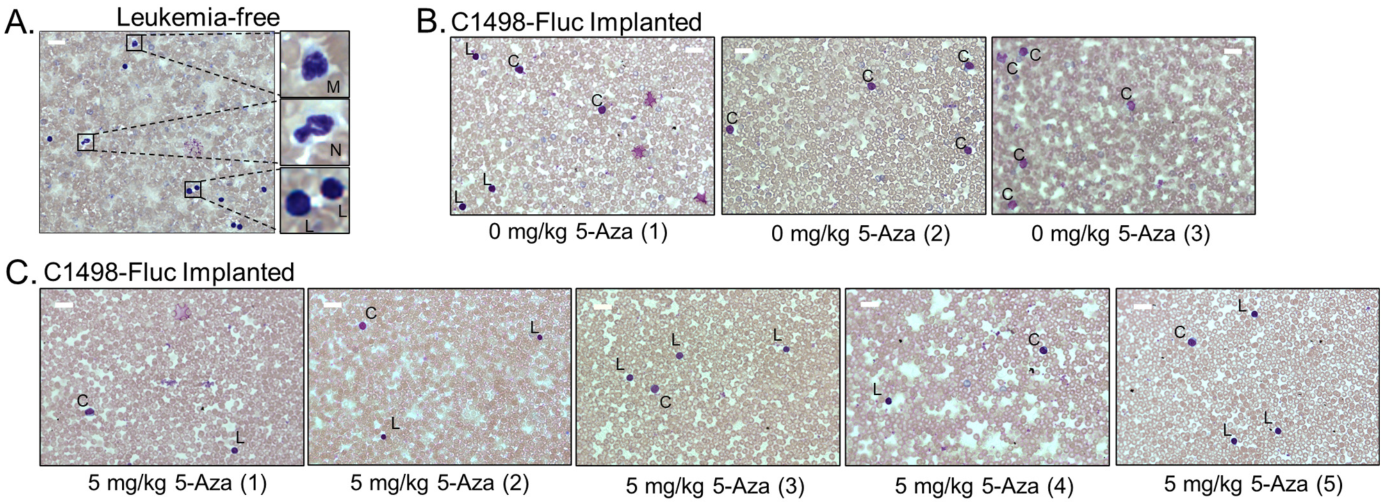

Figure S1. C1498-FLuc engraftment can be confirmed through blood smears prior to end stage of disease. Ten microliters of blood was smeared on a microscope slide, fixed in methanol, and stained with Wright's stain. Microscope images from un-engrafted, un-treated mice (A) show large numbers of lymphocytes (L) and few numbers of monocytes (M) and neutrophils (N) surrounded by abundant red blood cells consistent with normal percentages from peripheral mouse blood (insets show morphologies at increased magnification). (B) Blood smears stained with Wright's stain from individual C1498-Fluc (C) engrafted mice treated with vehicle (0 mg/kg 5-Aza) or (C) 5 mg/kg 5-Aza, 17 days post-challenge. Scale bar = 20μm.

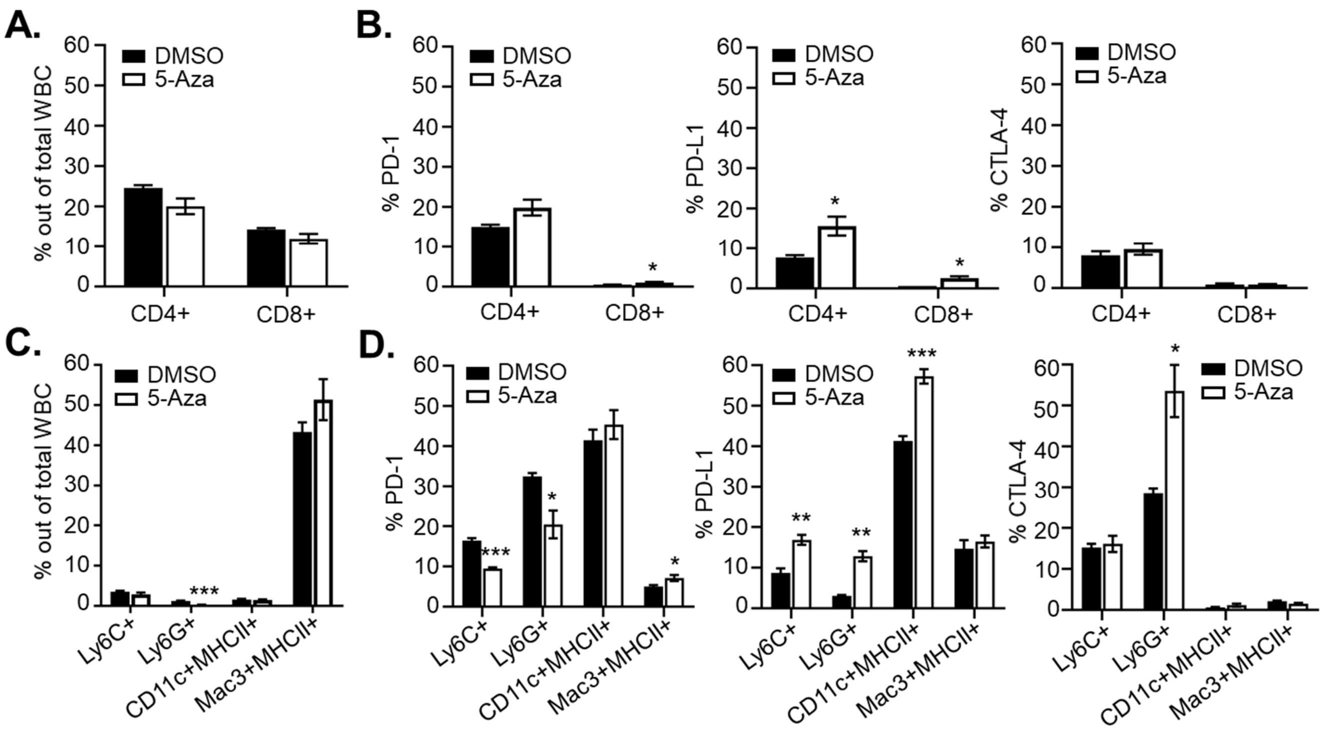

Figure S2. 5-Aza treatment alters the immune microenvironment in spleen. (A-D) C57Bl/6 mice were challenged with 8x104 C1498-FLuc cells via tail vein and AML was allowed to develop. On day 14 post-challenge, mice were given three consecutive, daily treatments of vehicle (DMSO) (n=4 mice) or 5 mg/kg 5-Aza (5-Aza) (n=5 mice). Forty-eight hours after the third treatment, spleen was processed for flow cytometry. Cells were surface stained with antibodies recognizing CD4, CD8, PD-1, PD-L1, and CTLA-4 (A, B). Percentages of positive cells analyzed by flow cytometry are shown out of gated lymphocytes. (E, F) Processed spleen cells were stained with antibodies recognizing Ly6C, Ly6G, CD11c, MHCII, Mac3 (intracellular), PD-1, PD-L1, and CTLA-4 (intracellular). Percentages of positive cells analyzed by flow cytometry are shown out of gated myeloid cells. For all panels (A-D) *$p<0.05$, **$p<0.01$, ***$p<0.001$, unpaired t-test with Welch's correction.
